# Energy Status and Body Composition Across a Collegiate Women’s Lacrosse Season

**DOI:** 10.3390/nu11020470

**Published:** 2019-02-23

**Authors:** Hannah A. Zabriskie, Bradley S. Currier, Patrick S. Harty, Richard A. Stecker, Andrew R. Jagim, Chad M. Kerksick

**Affiliations:** 1 Exercise and Performance Nutrition Laboratory, Department of Exercise Science, Lindenwood University, St. Charles, MO 63301, USA; hzabriskie@lindenwood.edu (H.A.Z.); bc129@lindenwood.edu (B.S.C.); pharty@lindenwood.edu (P.S.H.); rstecker@lindenwood.edu (R.A.S.); 2 Sport Medicine Research, Mayo Clinic Health Systems, Onalaska, WI 54650, USA; jagim.andrew@mayo.edu

**Keywords:** female athletes, energy balance, nutrition, RED-S, calories, recommendations, gender, health, energy availability

## Abstract

Little data is available regarding the energy and nutritional status of female collegiate team sport athletes. Twenty female NCAA Division II lacrosse athletes (mean ± SD: 20.4 ± 1.8 years; 68.8 ± 8.9 kg; 168.4 ± 6.6 cm; 27.9 ± 3% body fat) recorded dietary intake and wore a physical activity monitor over four consecutive days at five different time points (20 days total) during one academic year. Body composition, bone health, and resting metabolic rate were assessed in conjunction with wearing the monitor during off-season, pre-season, and season-play. Body fat percentage decreased slightly during the course of this study (*p* = 0.037). Total daily energy expenditure (TDEE) (*p* < 0.001) and activity energy expenditure (AEE) (*p* = 0.001) energy were found to change significantly over the course of the year, with pre-season training resulting in the highest energy expenditures (TDEE: 2789 ± 391 kcal/day; AEE: 1001 ± 267 kcal/day). Caloric (2124 ± 448 kcal/day), carbohydrate (3.6 ± 1.1 g/kg), and protein (1.2 ± 0.3 g/kg) intake did not change over the course of the year (*p* > 0.05). Athletes self-reported a moderate negative energy balance (366–719 kcal/day) and low energy availability (22.9–30.4 kcal/kg FFM) at each measurement period throughout the study. Reported caloric and macronutrient intake was low given the recorded energy expenditure and macronutrient intake recommendations for athletes. Athletic support staff should provide athletes with appropriate fueling strategies, particularly during pre-season training, to adequately meet energy demands.

## 1. Introduction

Energy is required for all bodily functions. The total amount of energy expended in one day, total daily energy expenditure (TDEE), is the sum of resting metabolic rate (RMR), activity energy expenditure (AEE), non-exercise activity thermogenesis (NEAT), and the thermic effect of food (TEF). While RMR is a consistent contributor to TDEE, making up about 60–65% of daily energy expenditure, AEE can vary widely from day to day within a single person and between different individuals [1,2]. In order to maintain energy balance, individuals must attempt to match energy intake with the amount of energy expended each day. Thus, regulation of energy balance is a primary focus of athletes and athletic professionals [3] to ensure optimal energy is available to support training, recovery, and lean body mass.

In instances when excess calories are consumed relative to TDEE, weight gain oftentimes occurs. Conversely, if insufficient calories are consumed, a state of negative energy balance exists during which athletes can experience undesirable loss of fat free mass (FFM) [4] and be at increased risk of injuries [5] and illness [6]. Energy availability, or the number of calories available to each kilogram of FFM after accounting for AEE is an emerging measure of energy status that is easier to assess, as opposed to TDEE or energy balance, from a time and logistics perspective, as it focuses solely on activity-related energy expenditure [7]. Low energy availability is correlated with a negative energy balance and Relative Energy Deficiency in Sport (RED-S) among female athletes [8]. RED-S is a comprehensive syndrome which includes the three components originally described as the female athlete triad [9] and extends to include a multifactorial state of physiological dysfunction that can have a profound impact on athlete health. It is important for athletes, coaches, athletic trainers, and all other athletic personnel to understand how to avoid these low energy states and to be aware of factors that may increase these risks.

Much of the research regarding athletic energy status has focused on male athletes. While efforts have established the energy status of female athletes performing endurance [8,10] and team sports [11,12,13,14,15,16], the generalizability of these results is hampered by the unique demands of each sport. Furthermore, the energy needs of an athlete can be dependent on sport type (e.g., aesthetic, continuous, and intermittent), position, level of competition, and seasonal training demands. For instance, in collegiate lacrosse, the non-stop clock, large field of play, and frequent changes in acceleration and direction create different energy requirements from those for athletes competing in other sports. These nuances are further complicated for athletes who are required to travel or perform frequently. Because of these distinctions, more research is needed to provide relevant information on energy requirements as it pertains to many types of female athletes.

Few researchers have undertaken the task of tracking energy status in a single cohort of female athletes over an entire annual training calendar. Reed et al. [13] has measured energy availability during pre-, mid-, and post-season periods in NCAA Division I female soccer players and Woodruff et al. [14] has compared energy availability from pre-season to post-season in elite female volleyball players. However, while these protocols assessed energy demands during or surrounding in-season play, these reports do not provide a long-term view of changes in energy status. Zanders et al. [17] has monitored collegiate (NCAA Division II) women’s basketball players longitudinally across a complete academic calendar (September–April); however, the demands facing basketball players are likely different from females participating in field-based team sports. Therefore, more research is needed to assess off-season energy status in addition to pre-season and in-season status in field-based team sports, as energy demands may differ due to variation in training practices and body composition goals. To meet these needs, the following investigation sought to document the fluctuations in energy expenditure, energy balance, and body composition over the course of an academic year in Division II collegiate female lacrosse players.

## 2. Materials and Methods

### 2.1. Research Design

**Figure 1:**
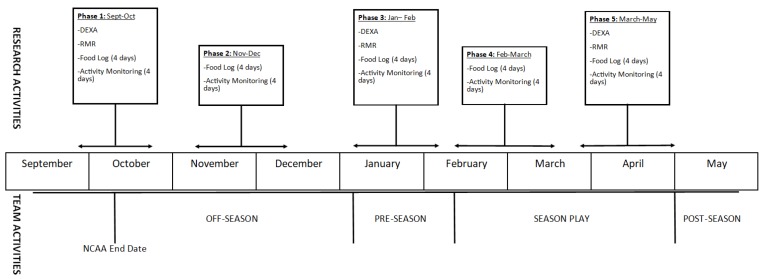
Overview of research and team activities. DEXA, dual energy X-ray absorptiometry; RMR, resting metabolic rate.

Data collection commenced at the beginning of the fall academic semester and finished at the end of the spring academic semester. Participants were observed and assessed for changes in RMR, body composition, bone density, energy intake, TDEE, and perceived recovery. Participants were assessed during five phases, with each testing period separated by four to six weeks. During each phase, participants were monitored for a period of four days (two weekdays, two weekend days), during which they wore a physical activity monitor (Actiheart, CamNTech, Cambridge, UK) and recorded all food consumed using a commercially available food and nutrition tracking application (MyFitnessPal, Under Armour, Baltimore, MD, USA). Monitoring of daily energy expenditure and energy intake occurred during all five phases. During the first, third, and fifth phases, body composition and bone density were assessed using dual energy X-ray absorptiometry (DEXA) scan and RMR was measured within two weeks of the monitoring period. Figure 1 provides an overview of each testing phase. Phase I consisted of practices and conditioning, with one group of six athletes participating in a game. Phase II consisted of off-season conditioning and captain-led practices. Phase III consisted of pre-season training and practices. Phase IV consisted of the first half of season-play and associated conditioning and practices. Phase V covered the second half of the season and finished just before the start of post-season tournament play.

### 2.2. Subjects

A total of 20 NCAA Division II female lacrosse athletes completed this investigation (mean ± SD: 20.4 ± 1.8 years; 68.8 ± 8.9 kg; 168.4 ± 6.6 cm; 27.9 ± 3% body fat). Athletes were recruited during a team meeting, during which researchers presented the study design and answered questions regarding participation. Initially, 22 NCAA athletes were recruited for this study, though any athlete who became injured and was unable to fully participate in team activities was excluded from participation. One athlete withdrew during Phase IV due to noncompliance (not wearing physical activity monitor) and another was excluded for medical reasons before the start of Phase III. The final sample included 20 athletes without significant injury who were fully participating in team activities during each assessment phase. All subjects were informed of the risks associated with participation in this study and signed an informed consent document approved by the Lindenwood University IRB (Protocol # IRB-19-L0012, approved 25 August 2017).

### 2.3. Methodology

#### 2.3.1. Anthropometrics, Body Composition and Bone Health

Body weight and height were measured during each phase of the study. Shoes and excess clothing were removed and then body weight was obtained to the nearest 0.1 kg (Digital Scale BWB-627A Class III, Tanita, Tokyo, Japan). Height was measured to the nearest 0.25 in using a stadiometer (HR-200, Tanita, Tokyo, Japan) and subsequently converted to cm.

Body composition was measured during phases I, III, and V. Participants arrived at the laboratory after following an overnight (8–10 h) fast and having abstained from exercise and caffeine for 24 h. Participants provided a urine sample to determine hydration status. In cases where the urine specific gravity was greater than 1.02, 24 fluid ounces of water were provided to achieve a more standardized hydration status for each testing period. A whole-body DEXA scan (Hologic Discovery A, Hologic, Bedford, MA, USA) was then performed to obtain body composition and bone density parameters using manufacturer provided software (Hologic APEX Software, Version 4.5.3, Hologic, Bedford, MA, USA) with the NHANES correction factor applied. However, it should be noted that only a full body scan was obtained and was used to calculate bone mineral content (BMC), bone mineral density (BMD), and Z-score.

#### 2.3.2. Resting Metabolic Rate

RMR assessment was performed during phases I, III, and V on the same day as the DEXA scan. A metabolic cart (TrueMax 2400 Metabolic Measurement System, ParvoMedics, Sandy, UT, USA) was calibrated to within less than 2% of the previous day’s calibration factor. All RMR assessments during Phase I were performed using the same cart. However, the laboratory purchased a second metabolic cart which was also used in phases III and V, and each athlete was assigned to one of the two carts for phases III and V. Therefore, half of study participants used the same cart for the entire study, while the other half used a different cart for two of the three phases.

Following completion of the DEXA and 15 min of quiet and seated rest, participants were directed to lay supine on a padded exam table. Participants then had a plastic hood and attached drape placed over their head and shoulders. A blanket was positioned over the participant to ensure a comfortable resting temperature and to eliminate any expired air from leaking out of the closed system. Gases were collected and analyzed for 20 min. RMR was calculated by identifying five consecutive min in the last 10 min of assessment with a less than 5% change in oxygen use. The average of these five min was considered to be the RMR (kcal/day).

#### 2.3.3. Diet Record

Diet records were kept during each phase for four consecutive days, which included two weekdays and two weekend days. Participants were asked to download the MyFitnessPal application to their mobile device and share their diary with a designated member of the study team. In one instance, the participant was unable to download the application and therefore kept a paper record during the day and uploaded her intake to the MyFitnessPal website each evening. Participants were educated on dietary reporting strategies by a trained research assistant. Study participants were provided educational materials about serving sizes (written and pictorial comparisons of common household items and respective serving sizes) and were instructed on how to alter serving sizes in the MyFitnessPal application. Further education about the functionality of the application was provided and participants were instructed to record all food and drink they consumed, including alcohol. Energy (kilocalorie) and macronutrients (carbohydrate, fat, and protein) data was retrieved from each participant’s diary for each phase and expressed as a daily average for total and relative intakes.

#### 2.3.4. Physical Activity Monitoring

Physical activity monitoring occurred during each phase of this study on the same days for which diet information was collected. Physical activity monitoring occurred on two weekdays and two weekend days which were consecutive. Each participant was outfitted with a physical activity monitor (Actiheart, CamNtech, Cambridge, UK) which was to be worn at all times, except when showering. Accelerometry data provided by the Actiheart device was used for all energy calculations within this study. The monitor was attached using two electrodes (Kendall 230 Foam Electrodes, Covidien, Mansfield, MA, USA), with one positioned over the xiphoid process and the other on the left side of the chest between and the fifth and sixth rib. When the monitor was initially applied by a study team member, each electrode location was outlined with permanent marker and participants were allowed to replace the electrodes as they lost adhesion. Additionally, participants were provided with an Actiheart-compatible chest strap (Polar, Kempele, Finland) to be used during heavy exercise instead of the electrodes.

The Actiheart devices have been previously shown to accurately calculate TDEE and AEE [18]. RMR was calculated via the manufacturer-provided software using the Schofield equation for use in the TDEE estimation. The software also automatically estimated TEF to be 10% of TDEE. Each of these values was recorded for each of the four monitoring days during each phase expressed as a daily average by collapsing the data collected during the four-day monitoring period.

Only six Actiheart devices were available for use during this study. Therefore, each phase took four weeks to complete, as the sample was divided into four groups of five to six athletes.

#### 2.3.5. Markers of Energy Deficiency

Energy balance (kcal/day) was calculated as the difference between energy intake and TDEE during each phase. Energy availability (kcal/kg FFM) was calculated during each phase using the following equation [19]:

(1) Energy Availability = (Energy intake – AEE)/kg FFM

For phases when FFM was not directly measured (i.e., for Phase II and Phase IV), the FFM measured in the previous phase was used.

RMR ratio, or the ratio of measured RMR to predicted RMR, was calculated per the findings of Staal et al. [20]. RMR ratio was calculated twice; it was calculated once using the Cunningham equation and once with the Schofield equation. The Cunningham equation has been utilized in prior research of RMR ratio [20] and has been validated in athletic populations [21]. The Schofield equation is [22]

(2) (RMR = [14.808 × weight (kg)] + 486.3)

It was also utilized for RMR ratio calculations due to it being the manufacturer default for RMR prediction in the Actiheart software. Fat-free mass index (FFMI) was calculated with no height correction as kilograms of FFM per meter of height squared (kg/m^2^). Both RMR ratio and FFMI were only calculated for phases when they were directly measured (Phases I, III, and V).

#### 2.3.6. Assessment of Recovery

During each phase, athletes were provided with a paper questionnaire featuring visual analog scales (VAS). Perceived rest, soreness, and training satisfaction were all assessed using VAS. Each VAS was 100 mm in length and responses are reported as a number between 0 and 100.

### 2.4. Statistical Analysis

All analysis was completed in SPSS v25 (Chicago, IL, USA). All data are presented as means ± SD. The Shapiro-Wilk test was used to identify non-normal data. Several of the body composition variables were moderately skewed, though transformations did not improve normality. Therefore, because the sample sizes were consistent throughout each phase, analysis was performed using a repeated measures ANOVA, relying upon the robustness of the procedure to overcome the moderate skew. Bonferroni *post hoc* corrections were utilized to identify significantly different phases, when indicated. Each ANOVA model was fit with *N* = 20 as that was the number of participants who completed every phase of the study. That was therefore the final sample size utilized for all results. Pearson correlations were used to quantify relationships between variables. A significance level of α = 0.05 was used.

## 3. Results

### 3.1. Body Composition and Bone Health

An overview of body composition and bone health for each phase is found in Table 1. This population of female lacrosse players had an average body mass index (BMI) of 24.23 ± 2.3 kg/m^2^ and an average weight of 69.3 ± 9.5 kg over the entire course of the study. The average percent body fat was 27.4 ± 3.0% throughout the year.

Weight (kg) did not significantly change over any of the five phases (*p* = 0.201). FM (*p* = 0.118) did not change between phases I, III, and V, while FFM trended toward a change (*p* = 0.054). However, body fat percentage decreased slightly over the year (*p* = 0.037), though Bonferroni *post hoc* testing only identified a trend (*p* = 0.076) towards athletes having significantly higher body fat percentage in Phase I compared to Phase V. Bone mineral content (*p* < 0.001) significantly increased over the season with Phase V being significantly greater than Phase I (*p* = 0.007; *p* = 0.028) and Phase 2 (*p* < 0.001; *p* = 0.014). However, bone mineral density did not significantly change (*p* = 0.17).

### 3.2. Metabolic Rate and Energy Expenditure

RMR was elevated (*p* < 0.001) during Phases III (*p* = 0.002) and V (*p* = 0.001) compared to Phase I. RMR was found to be moderately correlated with weight (*r* = 0.50–0.68, *p* < 0.05), FM (*r* = 0.48–0.67, *p* < 0.05), and FFM (*r* = 0.51–0.61, *p* < 0.05), during Phases I and III. Stronger correlations were noted between RMR and weight (*r* = 0.78, *p* < 0.001), FM (*r* = 0.78, *p* < 0.001), and FFM (*r* = 0.71, *p* < 0.001) in Phase V. When combining all phases, total body weight was most strongly correlated with RMR (*r* = 0.61, *p* < 0.001). A moderate correlation was also identified between RMR and FM (*r* = 0.58, *p* < 0.001) and FFM (*r* = 0.58, *p* < 0.001).

Energy expenditures from each phase can be found in Table 2. TDEE was significantly different during the phases of this study (*p* < 0.001), with Phase III resulting in greater EE than Phases I, IV, and V (*p* < 0.05). AEE changed significantly over the course of the season (*p* = 0.001), with AEE recorded in Phase III being significantly higher than that recorded during Phase I (*p* = 0.004), Phase IV (*p* = 0.002), and Phase V (*p* = 0.04), while trending towards being significantly greater than that in Phase II (*p* = 0.065). Physical activity level (PAL) was also significantly higher in Phase III than in any other phase (*p* < 0.05), as depicted in Table 2.

### 3.3. Energy Intake

Self-reported caloric intake, including food, beverages, and alcohol, did not significantly change over the season (*p* = 0.247), and the athletes recorded ingesting 2124 ± 448 kcals on average throughout all phases (see Table 3). The ingestion of both absolute (*p* = 0.262) and relative (*p* = 0.146) carbohydrate intake was constant across the year. The same was also true for absolute (*p* = 0.168) and relative (*p* = 0.163) protein intake. Absolute fat ingestion, however, did change during the five phases (*p* = 0.03); specifically, Phase II resulted in significantly lower absolute fat ingestion than Phase V.

### 3.4. Energy Balance, Energy Availability and Surrogate Markers of Energy Deficiency

As presented in Table 4, energy availability changed significantly during the course of the academic year (*p* = 0.017). *Post hoc* comparisons showed that Phase III trended toward a lower energy availability than in Phase I (*p* = 0.058) and Phase IV (*p* = 0.057). Energy balance also changed across the course of this investigation (*p* = 0.01), with Phase III trending towards a more negative energy balance than Phase I (*p* = 0.053) and significantly a poorer balance than Phase IV (*p* = 0.029).

RMR ratio was significantly different during the three phases in which it was assessed (*p* ≤ 0.001) for both the Schofield and Cunningham equations. RMR ratio was found to be lowest during Phase I, which was lower than both Phase III (*p* < 0.02) and Phase V (*p* = 0.001). FFMI did not significantly change over the season (*p* = 0.175), with the average FFMI being 16.6 ± 1.2 kg/m^2^. Only during Phase III was a significant correlation noted between RMR ratio (Cunningham equation) and energy availability (*r* = −0.445, *p* = 0.049). All other time points revealed no significant correlations between surrogate markers (both Schofield and Cunningham RMR ratios or FFMI) and energy balance or energy availability (*p* > 0.05).

### 3.5. Recovery Measures

Few relationships between energy balance, energy availability, and perceived measures of recovery were identified. Energy balance (*r* = 0.581, *p* = 0.007) and energy availability (*r* = 0.6, *p* = 0.005) were positively correlated with sleep quality during Phase II. During Phase V, energy balance (*r* = 0.514, *p* = 0.02) and energy availability (*r* = 0.496, *p* = 0.026) were found to be significantly positively correlated with perceived rest. In addition, energy availability was positively correlated with training satisfaction during Phase V (*r* = 0.45, *p* = 0.046).

## 4. Discussion

The purpose of this investigation was to assess the body composition, energy expenditure, and dietary habits of NCAA Division II female lacrosse players during an academic year. While body composition variables and diet were relatively stable, energy expenditure changed significantly during the course of the study. However, the athletes appeared to be in a consistent state of negative energy balance due to their self-reported energy intake. Energy availability was also shown to be low, hovering near the clinical energy deficiency threshold of 30 kcal/kg FFM [19]. It should be noted that objective measures of energy status such as body weight and body composition did not significantly change, which may offer evidence of some level of underreporting of dietary intake as has been previously reported.

While the athletes in the present study demonstrated a negative energy balance at every time point, this is not unprecedented. Hill et al. [10] has described low self-reported caloric intake resulting in significant energy deficiency in lightweight rowers, though it was noted that underreporting of food was problematic in this population. Also in agreement with our findings, negative energy balance and similar levels of TDEE have been reported in elite synchronized swimmers [11], junior elite female soccer players [12], and Division II female basketball players [17]. Additionally, a study recording only energy expenditure and not energy intake has identified similar levels of TDEE in elite female soccer players [15]. Despite the presented cohort having a significant energy deficiency and low energy availability with no significant changes in body composition, it is reasonable to suspect, based upon prior reports detailed above, that this is due to underreporting of dietary intakes. Food logs can present a substantial participant burden and, unfortunately, are frequently fraught with underreported and misreported information [23]. Thus, until more accessible and simpler methods of accurately recording dietary intake are available, collecting a valid representation of energy intake will continue to be a barrier in energy balance and energy availability investigations. The only diet-related variable that changed over the course of the season was absolute fat intake, and this change, specifically low intake during Phase II compared to the end of the season, is likely a reflection of schedule demands and food availability while travelling for competition during Phase V.

The greatest energy deficiency was reported during Phase III, which occurred during pre-season training immediately following the academic winter break. In accordance with our findings, previous studies have also reported the highest energy expenditure during pre-season preparation [13,17,24]. However, there was no concomitant increase in energy or macronutrient intake in Phase III. Fat intake during Phase V was increased in comparison to other phases. We are not able to specifically identify why fat intake was increased during this phase, but it is tempting to speculate that due to the rigors of in-season travel and classes, etc. that athletes were left with less time to consider their food choices and consequently selected foods that were higher in fat content. Altogether, these changes and suggestions create the need for athletic support staff to emphasize proper nutrition, and specifically greater caloric intake, while also focusing on how to become competition ready during pre-season training when risk of energy deficiency may be higher. Athletes may also need to be better educated regarding how to anticipate travel and training schedules that should require a greater energy intake.

The assessment of energy availability or energy balance can be challenging, as it relies upon accurate dietary records and participant compliance with energy expenditure estimation methods. Therefore, some alternative markers of energy deficiency or low energy availability have been suggested to offer insight into energy status while minimizing participant burden. One of these markers is the ratio of measured RMR to predicted RMR, with values less than 0.90 representing suppressed RMR and energy deficiency [20]. Because body mass and body composition are also influenced by energy status, some metrics of body composition have been advocated to offer insight into energy status. While low BMI is generally associated with RED-S [9,25], it may not be a sufficient surrogate marker as it fails to account for body composition. Unpublished data (currently in review) from our research group in a large cohort of nearly 400 female athletes has identified and proposed a lower limit of fat-free mass index as a simple way of estimating energy status with minimal testing required (DEXA only). In this paper, we proposed a lower limit FFMI value of 16.92 kg/m^2^ as evaluated by a whole-body DEXA scan utilizing the TBAR 1209 correction factor. Whether or not this value holds true against future investigation and whether or not this FFMI value is actually correlated to low energy availability remains to be seen. As reported, no correlation was noted between FFMI and energy balance or availability. However, a different correction factor (NHANES) was utilized. Results from the present study provide interesting insight into the use of surrogate markers of energy deficiency longitudinally. For example, there were no changes noted in RMR ratio or FFMI over the course of this study despite significant changes in energy deficiency between phases. In addition, no meaningful correlations were identified between RMR ratio or FFMI and energy balance or energy availability. However, both of these measures simply may not respond to changes in energy deficiency very rapidly, suggesting that these measures may be more appropriate for acute assessments of high-risk athletes, particularly if screening a large group of athletes at one time. The utility of RMR ratio is also dependent upon the use of accurate and appropriate prediction equations. Previous research has documented that many accepted equations do not accurately predict RMR in Division II female athletes [26]. The observed increase in RMR during Phase III and V without any change in calorie intake, body mass, or fat-free mass is challenging to interpret. While not fully assessed within our current paper, it is possible that some level of adaptive thermogenesis was occurring that resulted in RMR being elevated due to the increased energy expended secondary to the recovery demands of in-season activity. While possible, all RMR measures followed standardized protocols whereby each person had refrained from exercise for at least 24 h and observed an overnight fast. Therefore, it is not likely the change was due to a deviation in our measurement approach. In addition, it is also possible the activity levels outside of documented training activities may have occurred which may account for our reported changes in RMR. Nonetheless, future research should address these considerations.

Despite the robust scope of this investigation, it was not without limitations. One of the primary limitations was the lack of confirmatory data via questionnaires or logs, primarily with regards to menstrual function and physical activity. It was known, anecdotally, that it was not uncommon for this athletic team to participate in self-directed exercise in addition to scheduled team activities. Including a daily physical activity log would have aided in the validation of the AEE and TDEE measurements and would likely help to better explain participants’ energy balances. Administration of an assessment for disordered eating would have provided greater insight into RED-S in this population. Additionally, it would have been beneficial to monitor menstrual function. Tomten and colleagues [27] have published a study investigating weight stable runners with and without menstrual disorders. Despite similar energy expenditures and stable body weights, runners who exhibited menstrual dysfunction also tended to have lower energy intake and therefore also a negative energy balance. While the present study similarly evidenced weight stability despite an estimated energy deficit, menstrual function was not assessed. Hence, identifying athletes with menstrual irregularities could potentially explain how the body altered energy expenditure to accommodate the deficient energy intake, particularly considering the consistency of body weight measurements that were observed across our study design. Future studies should include both physical activity and menstrual function questionnaires.

## 5. Conclusions

Despite the limitations previously described, the scope of this study is unprecedented in female lacrosse athletes. This is the first investigation to have assessed energy expenditure, energy status, and body composition with this volume over an entire academic year in female athletes playing a field-based team sport. Overall, the female athletes in this study expended significant amounts of energy each day and consistently failed to match their levels of energy expenditure with adequate caloric intake. Because of this, athletes exhibited a negative energy balance and a low energy availability. Consequently, female team sport athletes should not be overlooked as a population at risk of negative energy balance and low energy availability. The results of this study also help to provide insight into PAL values of female team sport athletes which can be used by sport nutrition practitioners to identify energy requirements of comparable athletes undergoing similar training and potential changes throughout a season.

## Figures and Tables

**Table 1:** Body Composition and Bone Health over the Academic Year.

	Phase 1 (Off-Season)	Phase 2 (Off-Season)	Phase 3 (Pre-Season)	Phase 4 (In-Season)	Phase 5 (In-Season)	Overall *p*-Value
Body Weight (kg)	68.8 ± 8.9	69.6 ± 9.5	69.6 ± 10.0	69.3 ± 10.0	68.9 ± 10.1	0.20
FFM (kg)	47.0 ± 5.3	--	47.9 ± 5.4	--	47.4 ± 5.6	0.054
FM (kg)	18.4 ± 4.4	--	18.3 ± 4.7	--	17.8 ± 4.8	0.118
Percent Fat (%) *	27.9 ± 3.0	--	27.3 ± 2.7	--	27.0 ± 3.2	**0.037**
BMC (g) *	2575 ± 230 ^5^	--	2572 ± 230 ^5^	--	2610 ± 247 ^1,3^	**<0.001**
BMD (g/cm^3^)	1.20 ± 0.07	--	1.20 ± 0.07	--	1.24 ± 0.14	0.167
*z*-Score *	1.30 ± 0.76 ^5^	--	1.28 ± 0.80 ^5^	--	1.46 ± 0.75 ^1,3^	**0.004**

Data is presented as mean ± SD. Legend: BMC, bone mineral content; BMD, bone mineral density; FFM, fat-free mass; FM, fat mass. * Significant effect of Phase via Repeated Measures ANOVA. ^1^ Significantly different from Phase I. ^3^ Significantly different from Phase III. ^5^ Significantly different from Phase V. The bold indicates statistical significance.

**Table 2:** Resting Metabolism and Daily Energy Expenditure over the Academic Year.

	Phase 1 (Off-Season)	Phase 2 (Off-Season)	Phase 3 (Pre-Season)	Phase 4 (In-Season)	Phase 5 (In-Season)	Overall *p*-Value
RMR (kcal/day) *	1536 ± 152 ^3,5^	--	1683 ± 162 ^1^	--	1732 ± 244 ^1^	**<0.001**
TDEE (kcal/day) *	2608 ± 378 ^3^	2579 ± 376	2798 ± 391 ^1^	2513 ± 248 ^3^	2582 ± 303 ^3^	**<0.001**
AEE (kcal/day) *	842 ± 267 ^3^	804 ± 244	1001 ± 267 ^1^	749 ± 161 ^3^	817 ± 235 ^3^	**0.001**
PAL *	1.75 ± 0.19 ^3^	1.72 ± 0.14 ^3^	1.87 ± 0.15 ^1^	1.69 ± 0.15 ^3^	1.73 ± 0.18 ^3^	**0.001**

Data is presented as mean ± SD. Legend: AEE, activity energy expenditure; PAL, physical activity level; RMR, resting metabolic rate; TDEE, total daily energy expenditure. * Significant effect of Phase via Repeated Measures ANOVA. ^1^ Significantly different from Phase I. ^3^ Significantly different from Phase III. ^5^ Significantly different from Phase V. The bold indicates statistical significance.

**Table 3:** Self-Reported Caloric and Macronutrient Intake across the Academic Year.

	Phase 1 (Off-Season)	Phase 2 (Off-Season)	Phase 3 (Pre-Season)	Phase 4 (In-Season)	Phase 5 (In-Season)	Overall *p*-Value
Calories (kcal/day)	2242 ± 462	2015 ± 451	2079 ± 435	2124 ± 505	2161 ± 392	0.247
Carbohydrate (g/day)	262 ± 61	231 ± 59	247 ± 74	248 ± 66	236 ± 74	0.262
Protein (g/day)	80 ± 19	72 ± 20	82 ± 22	84 ± 16	79 ± 20	0.168
Fat (g/day) *	78 ± 20	70 ± 25 ^5^	74 ± 23	81 ± 26	88 ± 23 ^2^	**0.03**
Relative Carbohydrate (g/kg/day)	3.9 ± 1.1	3.4 ± 0.9	3.6 ± 1.2	3.6 ± 0.9	3.5 ± 1.2	0.146
Relative Protein (g/kg/day)	1.2 ± 0.3	1.1 ± 0.3	1.2 ± 0.4	1.2 ± 0.3	1.2 ± 0.4	0.163

Data is presented as mean ± SD. kcal = Kilocalorie; g = gram; kg = kilogram; * Significant effect of Phase via Repeated Measures ANOVA. ^2^ Significantly different from Phase II. ^5^ Significantly different from Phase V.

**Table 4:** Energy Balance, Energy Availability and Surrogate Markers of Energy Deficiency.

	Phase 1 (Off-Season)	Phase 2 (Off-Season)	Phase 3 (Pre-Season)	Phase 4 (In-Season)	Phase 5 (In-Season)	Overall *p*-Value
Energy Balance (kcal/day) *	−366 ± 527 ^‡^	−564 ± 484	−719 ± 440	−389 ± 432 ^3^	−421 ± 418	**0.01**
Energy Availability (kcal/kg FFM) *	30.4 ± 11.0 ^‡^	26.2 ± 10.5	22.9 ± 8.5	28.7 ± 9.5 ^‡^	28.9 ± 9.2	**0.017**
RMR Ratio, Schofield (Measured/Predicted) *	1.02 ± 0.7	--	1.11 ± 0.1 ^1^	--	1.15 ± 0.1 ^1^	**<0.001**
RMR Ratio, Cunningham (Measured/Predicted) *	1.0 ± 0.1	--	1.08 ± 0.1 ^1^	--	1.1 ± 0.1 ^1^	**0.001**
Free Mass Index (kg/m^2^)	16.6 ± 1.2	--	16.7 ± 1.2	--	16.5 ± 1.3	0.175

Data is presented as mean ± SD. kcal = Kilocalorie; g = gram; kg = kilogram; * Significant effect of Phase via Repeated Measures ANOVA. ^1^ Significantly different from Phase I. ^3^ Significantly different from Phase III. ^‡^ Trend toward difference from Phase III. The bold indicates statistical significance.
